# Classification Framework of the Bearing Faults of an Induction Motor Using Wavelet Scattering Transform-Based Features

**DOI:** 10.3390/s22228958

**Published:** 2022-11-19

**Authors:** Rafia Nishat Toma, Yangde Gao, Farzin Piltan, Kichang Im, Dongkoo Shon, Tae Hyun Yoon, Dae-Seung Yoo, Jong-Myon Kim

**Affiliations:** 1 Department of Electrical, Electronics and Computer Engineering, University of Ulsan, Ulsan 44610, Republic of Korea; 2 ICT Convergence Safety Research Center, University of Ulsan, Ulsan 44610, Republic of Korea; 3 Electronics and Telecommunications Research Institute (ETRI), Daejeon 34129, Republic of Korea; 4 PD Technologies Cooperation, Ulsan 44610, Republic of Korea

**Keywords:** artificial neural network, bearing fault diagnosis, condition monitoring, extreme gradient boosting, induction motor, motor current signal, random forest, wavelet scattering transform

## Abstract

In the machine learning and data science pipelines, feature extraction is considered the most crucial component according to researchers, where generating a discriminative feature matrix is the utmost challenging task to achieve high classification accuracy. Generally, the classical feature extraction techniques are sensitive to the noisy component of the signal and need more time for training. To deal with these issues, a comparatively new feature extraction technique, referred to as a wavelet scattering transform (WST) is utilized, and incorporated with ML classifiers to design a framework for bearing fault classification in this paper. The WST is a knowledge-based technique, and the structure is similar to the convolution neural network. This technique provides low-variance features of real-valued signals, which are usually necessary for classification tasks. These signals are resistant to signal deformation and preserve information at high frequencies. The current signal data from a publicly available dataset for three different bearing conditions are considered. By combining the scattering path coefficients, the decomposition coefficients from the 0th and 1st layers are considered as features. The experimental results demonstrate that WST-based features, when used with ensemble ML algorithms, could achieve more than 99% classification accuracy. The performance of ANN models with these features is similar. This work exhibits that utilizing WST coefficients for the motor current signal as features can improve the bearing fault classification accuracy when compared to other feature extraction approaches such as empirical wavelet transform (EWT), information fusion (IF), and wavelet packet decomposition (WPD). Thus, our proposed approach can be considered as an effective classification method for the fault diagnosis of rotating machinery.

## 1. Introduction

Induction motors (IMs) are widely used rotating machinery in the manufacturing and power industries, due to their certain advantages such as low cost, easy controlling mechanism, robust design, high efficiency, and reliability. However, the likelihood of faults cannot be overlooked, as the motors experience significant electrical and mechanical loads because of their prolonged working periods [1]. An intrinsic flaw in the machine or adverse surrounding conditions could be the reason for failure. If the initial erratic behavior is not identified, it can lead to motor failure, which will result in downtime and increased operation loss. Rotating machine condition monitoring has thus become increasingly interesting to researchers due to the inherent vulnerability to damage and failure of these machines. In order to improve the accuracy and capabilities of fault diagnosis systems, researchers are currently analyzing weak fault signals to extract fault features and classify them to enable real-time monitoring and diagnosis [2]. It is important to diagnose and monitor faults accurately and in a timely manner to prevent significant damage, extend the life of machines, increase accessibility, and lessen maintenance costs [3].

Depending on the components, IM faults can be classified as bearing faults, faults connected to the rotor, faults connected to the stator, etc. Among them, approximately 44% of these faults occur in bearings [4,5]. In the case of bearing faults, the damage can occur in any of the four main components: the inner race, the outer race, the balls, and the cage. However, 90% of faults occur in the inner and outer races [6].

In attempts to avoid dangerous accidents due to electric motor failure, breakdown maintenance methods were initially replaced by time-based preventive maintenance techniques. These were performed in accordance with working time periods, regardless of whether the machine needed a maintenance checkup or not. This approach is not only expensive but causes an interruption in the continuous working flow. Therefore, non-invasive condition-based maintenance techniques are currently considered to be more effective because they can reduce the amount of unnecessary scheduled preventive maintenance operations and lower the operation cost [7]. Numerous studies have been conducted on bearing fault diagnosis to develop new advanced approaches by utilizing innovative technologies and industrial equipment. Model-based [8] and data-driven [9] approaches are two basic techniques utilized in fault diagnosis. Model-based methods require precise modelling of the dynamics of a system with a comparatively small dataset, which is crucial to design approaches for highly nonlinear and ambiguous circumstances. On the other hand, data-driven approaches have become popular as data acquisition processes have become easier due to improvements in advanced sensor technology. A data-driven approach requires less engineering and design effort, and it is possible to extract useful information about a system’s current condition using modern feature engineering techniques [10].

Various types of sensor data are available for bearing fault diagnosis, such as vibration signals, acoustic emission signals, current signals, stray flux, thermal images, etc. [11]. Vibration signal-based analysis is a popular approach because of its high sensitivity to bearing faults, which can transmit any sudden change of intrinsic information immediately. The main limitation of using this type of signal is the high cost and high maintenance requirements of vibration sensors [12]. Fault analysis using acoustic emissions can be effective for early fault detection with a low-energy signal, but it requires a high amount of data to provide a good result, which increases the computational complexity of the overall method [13]. Motor current signals have been used to effectively diagnose electrical faults (broken rotor bar faults, stator winding faults) and bearing faults. The acquisition of the current signal does not require any external sensors, which reduces the overall installation and data collection costs of the system. Furthermore, current transducers can be used to measure the stator current from a single input source if frequency inverters and current transformers are not available. In addition to being highly reliable and noninvasive, motor current signal analysis (MCSA) is also considered one of the most effective condition monitoring methods in bearing fault diagnosis [14,15,16]. MCSA has been applied to both to analyze bearing faults and the fault severity in IMs with fault frequency analysis [1,17].

Generally, the original signal acquired from sensors is not enough to spot the existence of a fault and classify fault conditions, due to the presence of surrounding noise. To avoid ambiguity, extracting effective features from the sensor data by applying signal processing techniques is essential. There are diverse techniques for feature extraction. In fault diagnosis, time-domain features such as the rms, peak-to-peak, root mean square, etc., are calculated using statistical formulas on the sensor signal; frequency-domain feature extraction involves fast Fourier transform, envelope analysis, and high-order spectral analysis of the time-series signal [18]; and time-frequency domain features are derived using the wavelet transform, short-time Fourier analysis, Hilbert–Huang transform, etc. [6,19]. Based on the processing gain and the ability to separate the fault characteristic frequency from the noise, frequency-domain analysis can provide a better understanding of fault frequencies than time-domain analysis. However, in many cases, methods based on the frequency domain do not perform well with nonstationary signals, whereas time-frequency-based methods can be an effective approach to deal with both stationary and nonstationary types of signals [20].

The main drawback of the Fourier transform-based feature extraction process is that it becomes unstable at high frequencies. In such cases, the wavelet transform is considered an effective signal processing technique for fault classification of the rotation machinery [21,22,23]. To create time shift, the discrete wavelet transform (DWT) and the second-generation wavelet transform (SGWT) perform splitting or downshifting operations, which result in erroneous output due to the aliasing effect, which hampers reflection on the original state of the system [24]. Another variant of the wavelet transform, named dual-tree complex wavelet transform (DT-CWT), reduces the aliasing effect due to its time shift invariance and parity sampling properties. Although the wavelet transform is stable for signal deformation, this approach is not translation invariant when subsampling is involved. For these reasons, the Fourier, as well as wavelet transforms, cannot be considered as the ideal feature extractors.

Recently, a knowledge-based feature extraction technique has been developed by Bruna and Malat named wavelet scattering transform (WST), which utilizes complex wavelets to balance the discrimination ability and stability of the time-frequency domain signal [25]. This method filters the signal by assembling a cascade of wavelet decomposition coefficients, complex moduli, and low pass filtering operations. The WST approach enables the modulus and averaging operation of the wavelet coefficients to acquire stable features. After that, the cascaded wavelet transform is employed to recover the high-frequency information loss due to the previous wavelet coefficients’ averaging modulus operation. The resultant scattering coefficients possess local stability and translation invariance, and they have shown good performance in different application areas, such as image processing [26], sound classification [27], and heart sound classification [28]. The WST-based feature extraction process provides two advantages compared to other approaches in the fault diagnosis field. Firstly, the complex wavelet decompositions at multiple scales can provide rich descriptors of complicated structures for fault diagnosis through the co-occurrence of coefficients. Secondly, by using local weighted averaging, it is possible to reduce feature variability and preserve the local consistency of the class labels. It can also reduce the impact of noise originating from acquisition signals. Due to these reasons, researchers have become interested in this method and started implementing the WST in bearing and gearbox fault signal analysis. In [29], with the extracted scattering coefficients, a bearing fault was classified by SVM with 99% accuracy by utilizing vibration signals. Gearbox fault was analyzed in [30] with an acoustic emission signal by utilizing the WST with linear discriminant analysis (LDA); this approach had an affordable computational cost. Additionally, in [31], single and compound fault conditions were diagnosed by combining a denoising approach with WST coefficients to analyze rolling element bearings faults.

With the help of an effective feature extraction process, the original signal from sensors is transferred into a compact significant representation, which can be used as the input of machine learning classifiers for further training and optimizing decision functions. Common ML classifiers for fault diagnosis include support vector machine (SVM) [32], gradient boosting decision tree, k-nearest neighbors (KNN) [33], random forest (RF) [34,35], and neural network approaches [36,37]. Furthermore, deep learning (DL) methods have been implemented in multiple research areas, including bearing fault analysis, and provide very good performance [38,39]. Recently, unsupervised cross-domain diagnosis based on a joint transfer network [40] and modified auxiliary classifier GAN (MACGAN) [41] were implemented to generate multi-mode fault samples where the fault samples are limited.

To explore the ability to extract significant features of the WST, this paper aims to propose an accurate motor bearing fault classification framework based on the WST, two ensemble machine learning classifiers, and the artificial neural network (ANN). In scattering transform, the signal information can be scattered from one layer to another hierarchically, preventing information loss and maintaining signal stability. As the WST method operationally resembles deep CNN, it also divides the input data into multi-layer elements that contain both linear and nonlinear functions and have the advantages of deep CNN models [42]. The overall experiment was carried out with publicly available current signal data, and the resultant output was compared with some existing methods to validate the results.

Therefore, the main contributions of this study are as follows:

1. Investigate the applicability of the WST technique for extracting fault features to classify bearing states with ensemble ML algorithms and ANN.
2. The classification performance exhibits that the resulting coefficients can directly be used as features, thus no additional feature calculation step from the coefficients is required.
3. Resolve the feature extraction complexity of current signal-based bearing classification approaches due to their poor SNR and indirect measurement.

The rest of the paper is organized as follows. Section 2 presents the theoretical background related to this study. The experimental setup and a detailed description of the data are provided in Section 3. A detailed description of the proposed method and evaluation parameters are presented in Section 4. The experimental results using the proposed methodology and a comparison with existing papers on the same dataset are discussed in Section 5. Our conclusions are given in Section 6.

## 2. Theoretical Background

### 2.1. Bearing Fault Frequencies

**Figure 1:**
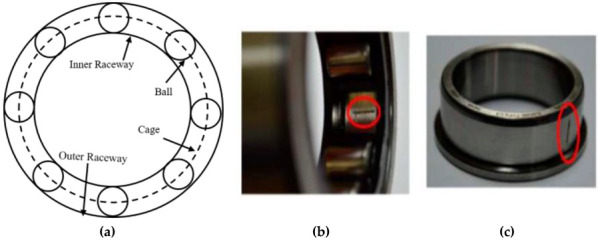
(**a**) Basic bearing geometric structure. Fault in the (**b**) outer race and (**c**) inner race.

Rolling element bearings (REBs) are thought to be the most important component of IMs because of their ability to lower friction and create a smooth rotating motion for a rotor to operate. The bearings serve as a holding component to ensure proper rotation from the shaft. They also allow for an electromechanical interaction between the stator and the rotor. The fundamental components of bearings are two different types of races, referred to as the inner and outer race, a set of rolling balls, and a cage in which each ball is enclosed by an identical distance. Numerous factors, such as excessive loading, improper installation, rotor misalignments, insufficient lubrication, and material fatigue, can cause bearing defects [43]. In general, the most frequent faults are those of a single element, such as faults in the outer race, inner race, or roller. However, multiple faults can also be produced simultaneously in different elements. In this work, the normal bearing condition and two faulty conditions (shown in Figure 1) are considered to investigate the bearing fault analysis with the motor current signal.

In general, every bearing component rotates at a fundamental frequency. Any time a fault arises during operation and the roller crosses the defect location during a rotation, a shock impulse at a specific defect frequency is produced because of the rise in vibration energy. Defect frequencies are the resultant frequencies of the defect signal based on the bearing element, and they can be calculated using the geometric parameters and rotational speed of the IM from the equations given in (1)–(3).


(1) $$\mathrm{Frequency of inner race fault:}\;F_{I}=\frac{N_{b}}{2}\times f_{m}\times \left(1+\left(\frac{D_{b}}{D_{c}}\times \cos\beta \right)\right)$$

(2) $$\mathrm{Frequency of outer race fault:}\;F_{O}=\frac{N_{b}}{2}\times f_{m}\times \left(1-\left(\frac{D_{b}}{D_{c}}\times \cos\beta \right)\right)$$

(3) $$\mathrm{Frequency of outer race fault:}\;F_{R}=\frac{D_{c}}{D_{b}}\times f_{m}\times \left(1-{\left(\frac{D_{b}}{D_{c}}\times \cos\beta \right)}^{2}\right)$$

Here, *N_b_* is the number of rolling components (balls), *D_b_* is the diameter of the ball, *D_c_* is the diameter of the cage, *β* is the load angle from the radial plane, and *f_m_* is the frequency of rotation.

Damage to the bearing causes the stator and rotor to move radially, which introduces characteristic fault frequencies into the current signals and causes oscillations. The stator and rotor are displaced radially by bearing problems, which affect the load torque and spinning eccentricity. As a result of changes in machine inductances, motor current signals experience amplitude, frequency, and phase modulation. With the phase angle *ϕ* and the angular velocity, the resultant current signal output due to a bearing fault can be written as shown by Equation (4),

(4) $$i\left(t\right)=\sum_{k=1}^{\infty }i_{k}\cos(\omega_{k}t+\phi )$$

and *ω_k_* is equivalent to $\frac{2\pi f_{\mathrm{bearing}}}{p}$.

Here, *f_bearing_* is the harmonic frequency, which can be written as $\left|f_{s}\pm mf_{v}\right|$, and *p* denotes the operating machine’s pole pair number. Furthermore, *m* and *f_s_* denote the harmonic index and supply frequency, respectively. However, *f_v_* can be expressed as either *f_inner_* or *f_outer_*.

The frequency auto search algorithm described in [44] can be used to calculate the estimated fault signature frequencies. Detecting bearing faults can be tricky because the harmonics generated by bearing failures might be close to or overlap with noise frequencies, making it difficult to tell them apart [45]. Therefore, it is challenging to find the bearing faults in an IM if the specifications of bearings are unknown or if the inverter frequency has fluctuated.

### 2.2. Wavelet Scattering Transform (WST)

A wavelet transform is a widely applied time-frequency analysis method that has the advantage of being stable and multi-scale in the presence of local deformation. It can effectively extract the local features from signals, but it is subject to change over time and can easily exclude significant signal features. A better time-frequency analysis technique built on the wavelet transform is the wavelet scattering transform (WST), which was proposed by Mallat [46]. The procedure is simply an iterative combination of a deep convolution network, consisting of low-pass filter averaging, a complex wavelet transform, and modulus operation [36]. With additional advantages of translation invariance, local deformation stability, and rich feature information representation, it also addresses the drawback of changing over time. For any given time-domain signal, *x*, the operation of WST can be described as follows:

1. At first, *x* is convolved with the dilated mother wavelet *ψ*, which has the center frequency of *λ*, to calculate the WST. This operation can be expressed as $x*\psi_{\lambda }$. Here, the average of the convolved signal, which oscillates at a scale of 2*j*, is zero.
2. After that, a nonlinear operator, such as a modulus, is applied to the convolved signal to eliminate these oscillations (i.e., $\left|x*\psi_{\lambda }\right|$). This procedure is used to make up for the information lost due to down sampling by doubling the frequency of the given signal.
3. Finally, a low-pass filter *φ* is applied to the resultant absolute convolved signal, which is equivalent to $\left|x*\psi_{\lambda }\right|*\varphi$

Therefore, for any scale (1≤j≤J), the first-order scattering coefficients are calculated as the average absolute amplitudes of wavelet coefficients over a half-overlapping time window having the size 2*j*. This can be written as (5):


(5) $$S_{1x}\left(t,\lambda_{1}\right)=\left|x*\psi_{\lambda_{1}}\right|*\varphi$$

The invariance ability will undoubtedly decrease when the high-frequency components are restored as a result of the aforementioned approach. By repeating the discussed steps on $\left|x*\psi_{\lambda_{1}}\right|$, the scattering coefficients for the second order can be calculated as (6):


(6) $$S_{2x}\left(t,\lambda_{1},\lambda_{2}\right)=||x*\psi_{\lambda_{1}}|*\psi_{\lambda_{2}}|*\varphi$$

The wavelet scattering coefficients for higher orders, where *m* ≥ 2, can be computed by iterating the mentioned process. This can be expressed as (7):


(7) $$S_{mx}\left(t,\lambda_{1},\lambda_{2},\ldots ,\lambda_{m}\right)=|||x*\psi_{\lambda_{1}}|*\psi_{\lambda_{2}}|\ldots \psi_{\lambda_{m}}|*\varphi$$

The resultant scattering coefficients can be found by accumulating all of the coefficient sets of the scattering transform generated from the *0*th to *m*th order, as shown in Equation (8) [25].


(8) $$S_{x}=\left\{S_{0x},S_{1x},\ldots ,S_{mx}\right\}$$

**Figure 2:**
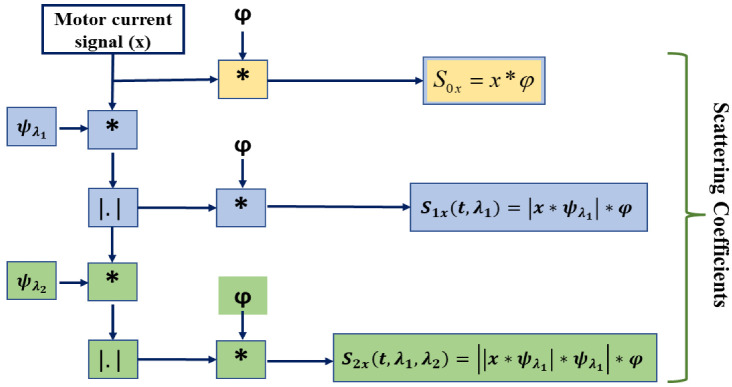
The schematic diagram of the feature extraction procedure with the second-order WST.

The basic steps of computing the wavelet scattering coefficients up to level 2 are illustrated in Figure 2. Here, the final feature matrix will be found by accumulating all the features from levels *S*_0*x*_,*S*_1*x*_,and *S*_2*x*_.

Here, *S*_0*x*_ represents the zero-order scattering coefficients, which evaluate the local translation invariance of the given input signal. The high-frequency components of the convolved signal are lost during each stage’s averaging operation, but they can be recovered in the following stage’s convolution operation with the wavelet. The WST method possesses the stability of time warp deformation, conversion in energy, and contraction, which makes the overall system robust in a noisy environment and appropriate for many classification tasks [30].

As a result of implementing the low-pass filter, φ, the network is invariant to translations up to a certain invariance scale. The resultant features from *S_x_* inherit properties of wavelet transforms, which make them stable against local deformations. This also allows the scattering decomposition to detect subtle changes in bearing signals’ amplitudes under different conditions and makes the classification task easier. Therefore, the wavelet scattering network can be used as an effective way to create robust representations of different bearing conditions that minimize the differences under the same condition and maintain enough discriminability to distinguish among different bearing conditions.

Despite the similarity in structure between wavelet scattering networks and CNNs, there exist two main differences: the filters are predetermined rather than learned, and the features are not just the outputs of the final convolution layer but are all the layers combined. Based on previous research, nearly 99% of the scattering coefficient energy is contained within the first two layers of the scattering coefficient, with the energy decreasing rapidly as the layer level increases [25,47]. The WST applied in this work also considers scattering coefficients for two orders, which are represented as *S*_1*x*_ and *S*_2*x*_. Through the cascaded wavelet decomposition, the WST can extract detailed feature information, and the local averaging technique can lessen the impact of noise. For these reasons, the WST can be considered a useful technique for extracting features in order to identify fault features in signals.

### 2.3. Feature Extraction Mechanism

In scattering transform, wavelet decomposition, modular operation, and low-pass filtering are employed to create invariant, stable, and informative signal representations. This process involves iterating over the input signal and calculating the wavelet modulus operator. The WST consists of different variables, such as the basis function of the selected mother wavelet (*ψ_λ_*), the *Q* factor, and the layer number of the scattering transform (*m*). Researchers found that, as long as the wavelet is complex, the outcome of the scattering transform is independent of the wavelet selection [46]. In the case of choosing the mother wavelet, Morlet (Gabor) wavelets were applied in this study. This wavelet can be expressed by Equation (9).


(9) $$\psi_{\sigma }\left(t\right)=c_{\sigma }\pi^{-0.25}e^{-0.5t^{2}}\left(e^{i\sigma t}-\kappa_{\sigma }\right)$$

Here, *κ_σ_* and *c_σ_* represent the admissibility criterion and normalization constant, respectively.

The quality factor (Q factor) defines how many filters are presented per octave. The selection of an effective Q factor requires expertise related to the spectral content of the considered signal. The Q value must be in the range of 1 to 32. The number of scattering coefficients and, thus, the dimensionality of the feature space are both increased as Q is increased. The dimensionality increases exponentially as the Q value increases when m > 1. It is desirable to keep Q as small as possible because an increase in its value does not improve the feature space’s ability to discriminate. A lower Q value also lowers the setup’s computational expense [48].

The number of layers in the scattering transform plays a crucial role in terms of performance and computational complexity. The selection of the number of layers is influenced by the fact that each layer must contain an adequate amount of energy for the succeeding levels to be useful. In different applications, scattering coefficients in the second layer are adequate, as the coefficients from the third layer do not help to improve the classification output [49]. Additionally, it is important to remember that the first layer creates an invariant by averaging the wavelet characteristics in a local area. After that, wavelets are used to collect the high-frequency information in the second layer to make up for the information loss caused by the low-pass filtering.

In the process of extracting features by implementing the built-in wavelet scattering network, the resultant feature output has three dimensions. This output can be expressed as *M* × *N* × *P*, where *M* is the scattering path, *N* is the wavelet scale, and *P* represents the signal number. As we will classify the features with ensemble classifiers, we need to convert this three-dimensional feature vector into two dimensions. Therefore, we multiply the values of *N* and *P* and reduce them to one dimension as *X* = *N* × *P*. Thus, the final two-dimensional feature vector will be *X* × *M*.

### 2.4. Classification with Ensemble Classifiers

Deciding which machine learning algorithm provides good classification performance is essential. Most recently published review articles contend that ensemble algorithms are superior to single prediction algorithms [50]. In many cases, any single algorithm cannot provide perfect prediction and good accuracy for any given classification problem, as each model has its own limitation in its working mechanism. By combining these types of models, which also refer to weak learners, it can be possible to boost overall accuracy. The ensemble learning technique uses several individual learners and a combination of strategies in order to achieve better results than each learner alone. The main objective of combining or ensembling models is to maximize the output from each model by reducing the model error and maintaining the model generalization. This technique helps to prevent the overfitting problem, and to reduce the bias and variance of the final model, thus the overall accuracy is increased [51]. Different ensemble techniques, such as bagging, boosting, stacking, and blending, are generally used to improve the aggregating model.

To classify IM faults with the wavelet scattering coefficient, two ensemble learning algorithms, i.e., random forest (RF) and Extreme Gradient Boosting (XGBoost), are used in this study, among them the RF implemented based on bagging mechanism and XGBoost executed based on boosting mechanism.

#### 2.4.1. Random Forest (RF)

**Figure 3:**
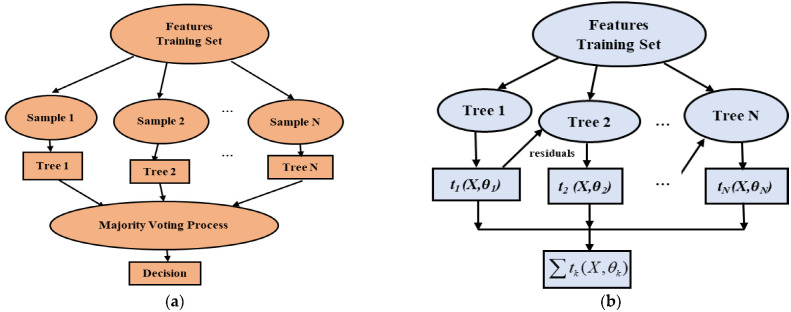
The basic architecture of (**a**) Random Forest and (**b**) Xtreme Gradient Boosting.

The random forest (RF) algorithm, introduced by L. Breiman [52], consists of a number of classification trees, each of which casts a single vote for the most common class to be given to the input data. The graphical representation of the RF algorithm is presented in Figure 3a. The class that receives the most votes is then chosen as the winner. RF is used for feature selection (FS), in addition to classification and regression tasks. The trees are created by combining datasets with bootstrap subsampling and various feature subsets for node-by-node splitting. Each tree has a distinct nature that, once mature, has little bias. Additionally, low correlation is attained by choosing random feature subsets for each tree. Finally, the RF algorithm yields low bias and low variance for the model after the assembling of all the trees. For individual trees in RF, bootstrap aggregating from bagging is intended to boost stability and accuracy [34]. In a classification problem, the class that receives the majority of votes from the trees is chosen for decision-making. Regression models, on the other hand, consider the mean of all predicted values from all decision trees. One of the key issues with a decision tree algorithm is overfitting, which the RF algorithm can also resolve. To decide the final output, RF employs a bagging technique in which a different random subset of features is used each time to train a single decision tree. As a result, RF is less likely to lead to overfitting.

Additionally, by employing a GridSearch technique, the parameter adjustment aids RF in overcoming the overfitting issue; this technique is also applied in this work. Several characteristics govern the diversity of the tree in the RF. A greater feature count guarantees the most highly correlated trees at the expense of high computing power, while a lower feature count produces a lack of correlation [23]. The number of trees, number of features in each split, maximum depth, and number of sample leaf nodes are parameters that must be considered to implement the RF algorithm. In general, both the classification and regression tasks require a large number of trees in order to reach a steady state. The RF model involves a splitting procedure that divides a single node into two or more nodes, with the model’s ultimate output determined by a majority voting method.

#### 2.4.2. Extreme Gradient Boosting (XGBoost)

An improved implementation of the gradient boosting decision tree (GBDT) method is known as Extreme Gradient Boosting (XGB). The GBDT uses only the first derivative, whereas XGBoosting often applies the first and second derivatives during optimization. Boosting is a method by which the ensemble aids in the fusion of several weak learners to create a single strong learner. This approach uses a sequential learning process where the current regression tree is further changed using the model to update the residuals to better suit the residuals (errors) from the previous tree. This is a constant learning process that progresses gradually to produce good results. Thus, newer regression trees trend to a maximum connection to the negative gradient of the loss function, which not only increases the algorithm’s adaptability but also converges on the loss function. For any given input, *x_i_*, the predicted output *ŷ_i_* for the GBDT can be written as shown in Equation (10) [53]:


(10) $${\hat{y}}_{i}=\phi \left(x_{i}\right)=\sum_{k=1}^{K}t_{k}\left(x_{i}\right),\;\;\;\;\;\;\;\;t_{k}\in T.$$

Here, *K* represents the function number in the given function space *T*.

These functions are introduced in XGB as a parameter, allowing the trainer to find functions *t_k_* that fit the data extremely well while training and identifying the regions accordingly. Therefore, XGB adds the regularization factor *Ω*(*t_k_*) to reflect the complexity of the tree, and it uses Equation (11) to define the objective function of the optimization in the training model.


(11) $$L\left(\phi \right)=\sum_{i}l\left({\hat{y}}_{i},y_{i}\right)+\sum_{k}\Omega \left(t_{k}\right)$$

Here, *ϕ* represents the model parameter. The loss function, which measures the degree of similarity between the training set and the model, can either be a logistic loss or square loss. The basic architecture of XGB is presented in Figure 3b. Another unavoidable aspect of XGB is the shared-memory multiprocessing API known as OpenMP, which facilitates the effective use of all CPU cores in parallel and the declaration of independent variables at the beginning of the training process, thereby reducing training complexity and computation time. Overfitting is typically more successfully resisted by the XGB model’s basic design.

### 2.5. Classification with Artificial Neural Network

The artificial neural network (ANN) is an information processing model constructed by stacking layers of perceptions or artificial neurons that have been inspired by our biological neural system. The ANN model can be used to learn complex patterns from data to solve classification and prediction problems. If ANNs are provided with relevant data, they can learn and model very complex and non-linear relationships in the data. Additionally, once they learn patterns, they can generalize their behavior, e.g., they can predict the future output for unseen data instances [54].

**Figure 4:**
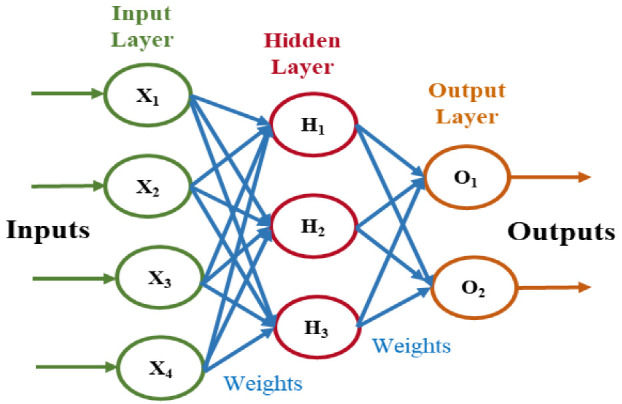
The basic architecture of the ANN model.

Figure 4 represents the architecture of a neural network with a single hidden layer. The first layer is the input layer, through which data are provided to the network. Each neural network has only one input layer. The data propagate through the hidden layers to the final layer, which is called the output layer.

The operation of the ANN model can be described by the step-by-step procedure as follows:

- Step 1: Initialize weights and bias and perform forward propagation

Every single unit in the hidden layer, called a perceptron, has weights and biases, and the perceptron performs a defined mathematical operation. For each input instance, the input is multiplied by the corresponding weight of the perceptron and the bias is added with it. Initially, the weight and bias values are randomly selected. If the input data are represented as *X* and the corresponding weight of the network is represented as *W*, then this operation can be mathematically represented by the Equation (12),


(12) $$Z^{l}=W^{l}X+b^{l}$$

Here, *l* represents the corresponding layer in the network. The next operation performed by the perceptron is passing the above result to a non-linear activation function in order to produce the output *y^l^*, as shown by the Equation (13).


(13) $$y^{l}=\sigma \left(Z^{l}\right)$$

Here, σ represents the activation function. This propagation of input data to the hidden layer and finally onto the output layer is called forward propagation.

- Step 2: Estimating error values

Then, the output of the network is compared with the actual output for the input observation to calculate the error, based on which weights of the neural network are updated. Updating the weights for minimizing the error or loss between the actual output and model-predicted output is referred to as training the network.

- Step 3: Performing backpropagation

Backpropagation is all about finding the impact of weights and biases in the resulting loss or error. The loss is generally calculated on a batch of input instances based on an appropriate cost function. The change in the cost function with respect to the weights and biases is determined by calculating the gradients of the cost function for all weights and biases.

- Step 4: Update Parameters

Once the gradient is computed, an optimization step is used to select the appropriate values for the weights and biases to minimize the prediction error. Gradient descent is generally used as the optimization algorithm.

The process mentioned from step 1 to step 4 is repeated until the loss is minimized to a satisfactory value; in other words, the network predicts an output that is very close to the actual output. A three-layer ANN model was implemented in this work, and the model parameters are listed in Table 1.

## 3. Experimental Testbed and Data Description

**Figure 5:**
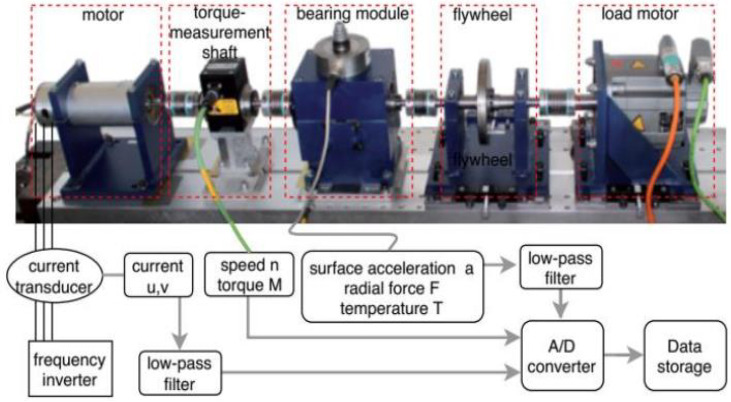
The test rig of the KAT-bearing data center.

The current signals of bearings from an IM used in this work were collected by the Research Center of Mechanical Engineering at the Paderborn University Kat Data Center, Germany [55]. The designed testbed consists of an induction motor, a measurement shaft, a test bearing module, a flywheel, and a load motor (Figure 5), and the testbed collects vibration, current, torque, temperature, and speed data synchronously with five different sensors [56]. A conventional industrial inverter was used to control the 425-W synchronous motor, which had a 16 kHz switching frequency. Two different phases of the current signal were recorded by the current transducer (LEM CKSR 15-NP model). Finally, the signal was filtered with a low-pass filter of 25 kHz and sampled at a rate of 64 kHz to transform it into a digital signal.

A total of 32 different test bearings were used in the data acquisition phase, including six normal bearings, 12 faulty bearings with artificially created damage, and 14 defective bearings with accelerated lifetime testing. The normal bearings were tested with various run-in times ranging from 1 h to more than 50 h. By following the VD1 3832 (2013) standard, the geometric sizes of the bearing defects were assigned in this testbed to create artificial damage. For the accelerated lifetime test, the inner race and outer race defects were introduced using plastic deformation damage, pitting damage, and fatigue damage techniques. The data collection process becomes more reliable when faults are injected into bearings while maintaining the requirements set out in ISO/IEC 15,243(2010) in terms of selecting fault measurements, such as the bearing geometry, fault location, and damage size. Additionally, a variety of defects with a wide range of severity levels were tested repeatedly under varied operating conditions by changing the rotational speed, load torque, and radial forces to make the overall data collecting procedure robust and reliable. The operating conditions used in the experimental process are listed in Table 2.

In this study, data from 17 bearings under three different conditions were considered among the 32 different bearing signals. The damage was single-point damage without any repetition or combination with other faults, and it was created artificially in the faulty bearings. Each of the bearings mentioned in Table 3 has 20 measurements, and each measurement contains a recording of 4 s. To conduct our analysis, we considered 1360 samples, each of which contains 1 s of data. As the sampling rate is 64 kHz, the final dimension of the dataset is thus 1360 × 64,000. The 1360 samples include observations of three conditions: normal, fault in the outer race, and fault in the inner race of the bearings. To perform the supervised classification task, we created three groups based on the bearing conditions; here, we labelled the normal condition as 0, the inner race fault as 1, and the outer race fault as 2.

**Figure 6:**
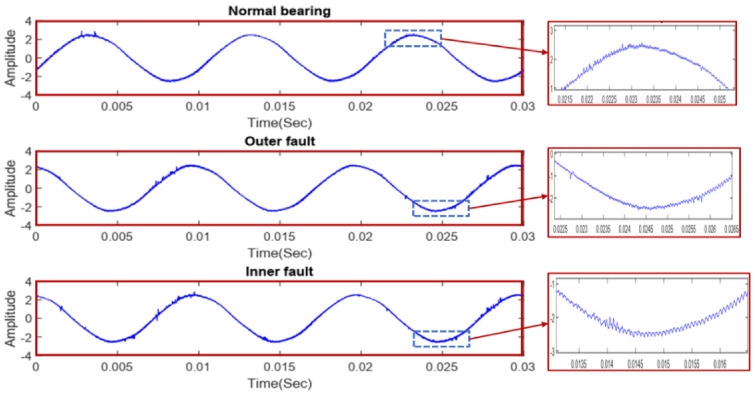
The time−domain representation of the three considered bearing conditions.

Figure 6 shows representations of the current signals in the time domain for the three different scenarios, where the signals show minor variations when observed in a zoomed-in view.

## 4. Proposed Method

**Figure 7:**
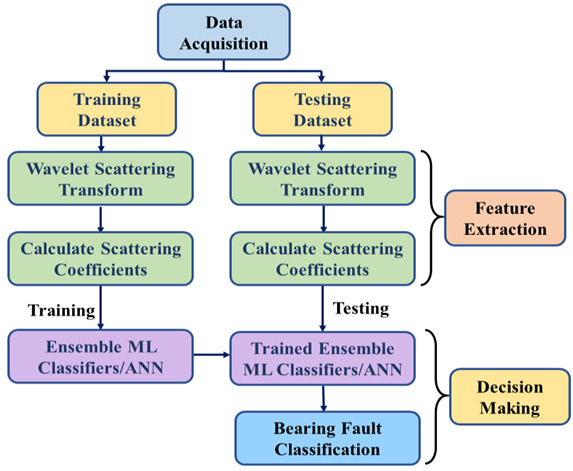
The fault classification method based on the WST and ensemble ML classifiers.

A framework for bearing fault classification with the motor current signal is illustrated in Figure 7. The bearing data for three different conditions, including normal conditions and two faulty conditions, are considered to form the KAT dataset mentioned in Section 2. The overall method is split into multiple phases, including data collection, pre-processing of the current signal data, feature extraction with the WST, training the two ensemble ML classifiers and ANN, and evaluating the model performance.

The collected current signal contains 4 s of data for each bearing condition and has a sampling frequency of 64 kHz. We consider samples with 1 s of data, providing 64,000 points for the three mentioned bearing conditions. The final input data matrix is 1360 × 64,000, where the matrix size for normal and outer fault conditions is 480 × 64,000 each and the dimension of the outer fault data is 400 × 64,000.

The input current signal data are divided into training sets and testing sets with a ratio of 80:20. We implement the overall process in MATLAB 2020. After finalizing the training and test data matrix, we need to build the wavelet scattering network according to the signal properties. A two-layer scattering network (m = 2) is utilized with a Q factor of [8 1].

**Figure 8:**
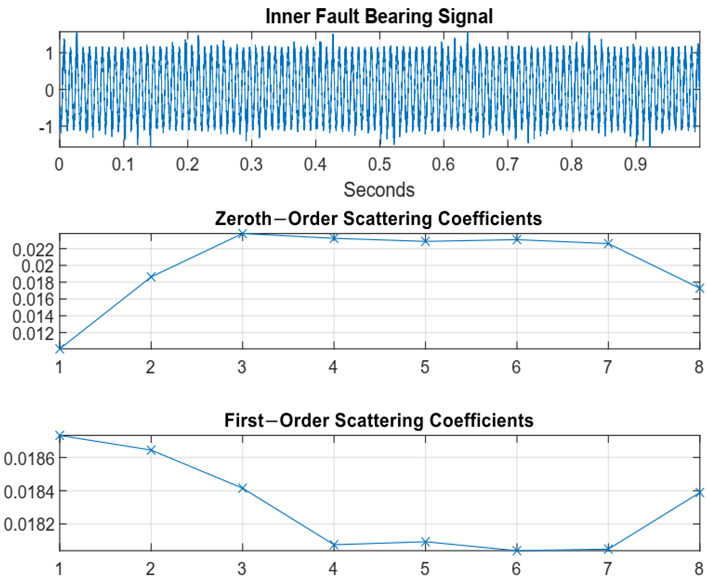
Time series bearing data, 0th and 1st order scattering coefficients of the inner race faulty conditions.

The 0th-channel represents the original signal, and the final scattering coefficients are generated through the following channels. The input bearing signal for the inner fault bearing condition and the corresponding 0th-order and 1st-order scattering coefficients are provided in Figure 8.

**Figure 9:**
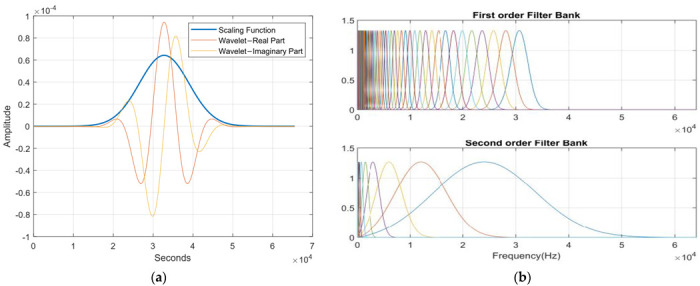
(**a**) The Morlet wavelet and its low−pass filter with a scaling function. (**b**) Frequency response of the first and second filter banks with eight and one wavelets per octave, respectively.

For wavelet decomposition, the Morlet wavelet is used, and the invariance scale value is fixed to 0.5 s. The basic wavelet and the designed two-layer wavelet scattering network with Q_1_ = 8 and Q_2_ = 1 are presented in Figure 9. This architecture preserved the most signal information for classification, as compared to other settings, for the invariance scale and wavelet octave resolution.

After applying the feature engineering techniques described in Section 2.3, the feature vector is generated from the training and testing data individually. This extraction process generates a set of features having a dimension of 499 × 8 for each row. Thus, the feature size depends on the length of the input signal, and the feature matrix dimension becomes 8704 × 499 and 2176 × 499 for the training and testing data, respectively. Finally, the ensemble models and ANN are trained with the training feature set, and the models’ performances with the test features extracted by the WST are evaluated.

### Fault Classification Performance Evaluation Parameters

According to the workflow provided in Figure 7, the bearing data are split into training and test sets. After extracting wavelet scattering coefficients from both sets of data, the classifier models are trained with the training coefficients. Finally, the test set is evaluated and the model evaluation parameters, such as the precision, recall rate, *F*1_*score*, and accuracy, are calculated. All these parameters can be obtained from the entries of the confusion matrix, which reflects how well the algorithm classified each record and where misclassification occurred. The training dataset’s actual labels are represented in the matrix’s rows, and the predictions are shown in the matrix’s columns. These evaluation parameters can be calculated using Equations (14)–(17).


(14) $$\mathrm{Precision}=\frac{TP}{TP+FP}$$

(15) $$F1\_\mathrm{score}=\frac{2\times \mathrm{Precision}\times \;\mathrm{Recall}}{\mathrm{Precision}+\mathrm{Recall}}$$

(16) $$\mathrm{Recall}=\frac{TP}{TP+FN}$$

(17) $$\mathrm{Accuracy}=\frac{TP+TN}{TP+FP+TN+FN}$$

## 5. Results and Discussion

The WST feature matrix is used with two ensemble classifiers (RF and XGB) and a multi-layer ANN model to assess the fault classification performance. To achieve the best performance from the mentioned algorithms, the optimal hyperparameters were decided by an intensive hyperparameter search with a wide range of parameter values. To determine the best set of hyperparameters, each independent set is applied to the model with k-fold cross-validation, and then the hyperparameter with the best fit is determined by using GridSearchCV (a scikit-learn class). For each classifier, grid search was conducted using 10-fold cross-validation to ensure the reliability of the resultant output, and the *R*^2^ metric was used to optimize model performance. The ranges of parameters considered, along with the optimum values, are listed in Table 4. All the associated programs are executed in a desktop computer equipped with an Intel(R) Core (TM) i7-9700 CPU @3.6 GHz, and 16 GB RAM.

**Figure 10:**
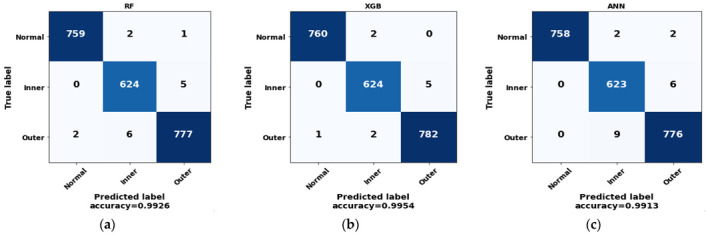
Confusion matrix of (**a**) RF, (**b**) XGB classifier, and (**c**) ANN.

After finishing the training process with the training data using the optimum value of each model, the model performance was finally tested with the test feature set. Along with the three mentioned models, we also trained and tested other familiar ML classifiers, including a support vector machine (SVM) and k-nearest neighbors (KNN), to compare the model performance. The evaluation parameters of these mentioned models and the confusion matrix of the three best-performing models (RF, XGB, and ANN) are presented in Table 5 and Figure 10, respectively.

**Figure 11:**
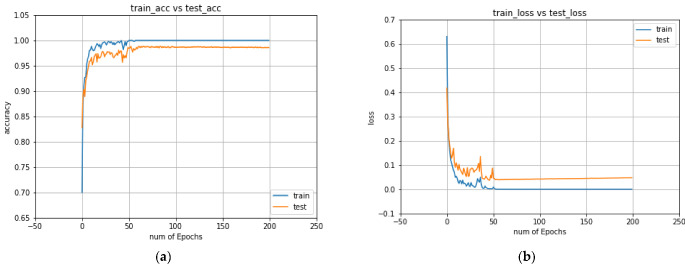
The accuracy (**a**) and loss curves (**b**) of the ANN model.

As can be seen from the confusion matrices, all three classifiers classify faults very accurately, with a negligible number of false positives and false negatives. The accuracy and loss curve for the ANN (up to 200 epochs) are provided in Figure 11. The accuracy curve indicates that the ANN model achieves a training accuracy of almost 100% and a testing accuracy of around 99%. After finishing 50 epochs, the model starts providing stable accuracy values until the final epoch of the training process.

**Figure 12:**
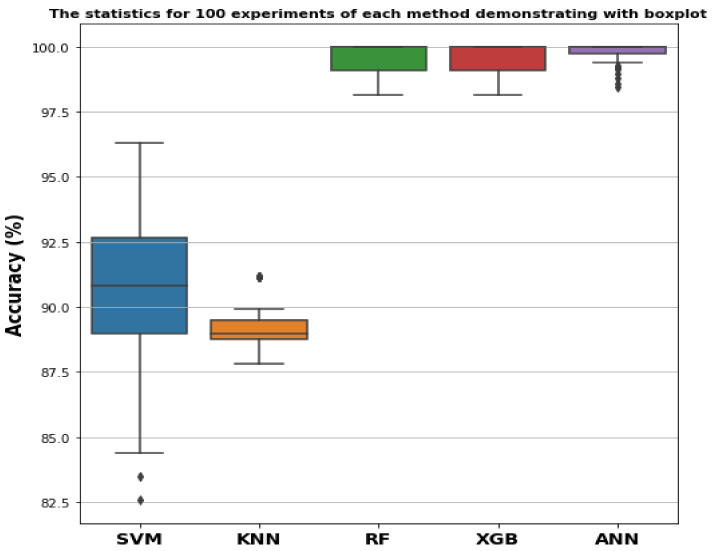
The boxplot represents the accuracy matrix of over 100 experiments for the SVM, KNN, RF, XGB, and ANN models.

For all the classifiers considered in this work, we have shown box plots of the accuracy distribution resulting from 100 experiments (Figure 12) to observe the stability and repeatability of our proposed model. For the SVM classifier, the achieved accuracy is around 92%, but the boxplot is wider than the other techniques and contains a long whisker laying toward the outlier of nearly 84%, which makes this method less stable. The KNN classifier can classify the bearing states with almost 90% accuracy and has a less wide boxplot than that of the SVM model. The accuracy scores of the corresponding boxplots for RF, XGB, and ANN were more than 99% and did not deviate that much from the mean and median values during the overall experiments. Based on the results presented in the boxplot, the extracted features with the WST can be classified with ensemble ML classifiers or ANN with high classification accuracy and stability.

### Comparison with Other Works

**Figure 13:**
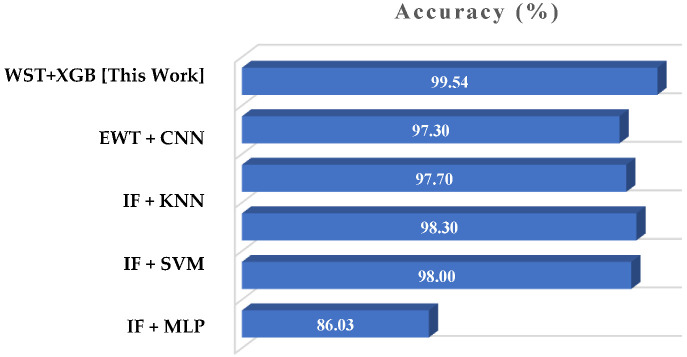
Comparison of the classification accuracy of designed model with existing works: (i) EWT + CNN [58], (ii) IF + KNN [57], (iii) IF + SVM [57], (iv) IF + MLP [57], and (v) WPD + SVM-PSO [55].

We conducted a comparative analysis with existing research works where the same current signal of the bearing conditions was considered. In [55], a three-level wavelet packet transform is applied for extracting significant features; 86.03% classification accuracy was achieved with the SVM-particle swarm optimization method. An information fusion-based fault classification approach was carried out by Hoang and Kang [57]. They replaced the combined time series data with greyscale images and classified the resultant images with three different supervised algorithms. The classification accuracy achieved by multilayer perceptron, KNN, and SVM approaches was 98%, 97.7%, and 98.3%, respectively. Furthermore, Hsueh et al. [58] applied the empirical wavelet transform technique to generate a greyscale image, achieving 97.3% accuracy by classifying it with the CNN model. Though the bearing current signal data of different conditions are very difficult to differentiate, researchers proved the image conversion technique can be an alternative way to generate distinguishable patterns and classify them easily with different ML algorithms. With our designed classification approach that combines WST and XGB models, we achieved 99.54% accuracy. The comparison outcomes are displayed in Figure 13.

By considering the outcomes of the discussed methods, it can be said that our proposed fault classification method based on WST features provides more than 99% accuracy with only feature extraction and classification steps. This technique not only reduces the complexity of the overall model, but it also does not require any feature selection steps to improve the model performance.

## 6. Conclusions

This paper presented a fault diagnosis approach for analyzing bearing faults using wavelet scattering transform-based features and ML classifiers. Modern industrial applications still place a great deal of importance on automatic fault detection and diagnosis via electrical signature analysis. For this reason, we utilized motor current signals from a publicly available bearing dataset to evaluate the proposed method. A two-layer WST was applied to the original signal to extract features in terms of the scattering coefficients and further train two ensemble classifiers (RF and XGB) and a multi-layer ANN. All three of these models perform very well with the WST features and achieve more than 99% accuracy along with low computational complexity. We included different operating conditions data for three different bearing conditions to validate the outcomes of our proposed model. Although we utilized an existing technique, the wavelet scattering transform, for generating features, this work shows that if WST-based features are used with ensemble classifier and ANN it could improve fault classification performance compared to EWT, IF, and WPD-based features for the same dataset. In this study, we considered only the classification of the bearing states; fault severity analysis was not considered. In our future research, we will consider fault severity analysis and incorporate more faulty conditions data from multiple sensors to provide a complete solution in the field of bearing fault diagnosis.

## Figures and Tables

**Table 1:** Layer-wise parameters of the designed ANN model.

Layer Type	Shape of the Output	Numbers of Parameters
dense_1	(None, 256)	128,000
dense_2	(None, 128)	32,896
dense_3	(None, 32)	8256
dense_4	(None, 3)	195
Total params: 169,347	Trainable params: 169,347	Non-trainable params: 0

**Table 2:** Working conditions of the KAT-bearing testbed.

Serial No	Rotating Speed (S) [rpm]	Radial Force (F) [N]	Load Torque (M) [Nm]
1	1500	1000	0.7
2	1500	1000	0.1
3	900	1000	0.7
4	1500	400	0.7

**Table 3:** Bearing conditions, bearing codes, and class labels for fault analysis.

Bearing Conditions	Bearing Code	Class Label
Normal Bearing	K001, K002, K003, K004, K005, K006	0
Outer Ring	KA04, KA15, KA16, KA22, KA30	1
Inner Ring	KI04, KI14, KI16, KI17, KI18, KI21	2

**Table 4:** Parameter selection through grid search.

RF			XGB			ANN		
Model Parameters	Considered Range	Optimum Value	Model Parameters	Considered Range	Optimum Value	Model Parameters	Considered Range	Optimum Value
Minimum sample leaf	(1, 2, 3)	3	Maximum depth	1 to 20	15	Number of epochs	(20, 50, 100, 200, 250]	200
Minimum sample split	(2, 4, 8, 16)	8	Gamma	0.1 to 1	1	Batch size	(32, 64, 128, 256)	32
Number of estimator	(20, 30, 50, 100, 150, 200, 250)	150	Number of estimator	50 to 1000	500	Learning rate	(0.001, 0.01, 0.1, 0.2, 0.3)	0.2
Maximum features	(3, 5, 7, 9)	3	Learning rate	0.1 to 1	0.1	Momentum	(0.0, 0.2, 0.4, 0.6, 0.8, 0.9)	0.9

**Table 5:** The resultant evaluation parameters.

	Precision	Recall	F1_score	Accuracy (%)
SVM	0.92	0.90	0.92	92.88
KNN	0.89	0.86	0.88	89.89
RF	0.99	0.99	1.00	99.26
XGB	0.99	1.00	0.99	99.54
ANN	0.99	0.99	0.99	99.13

## Data Availability

The data is publicly available.
